# Integrative Medicine Across the Pediatric Cancer Care Trajectory: A Narrative Review

**DOI:** 10.1007/s11912-024-01538-1

**Published:** 2024-05-11

**Authors:** Eran Ben-Arye, Noah Samuels, Georg Seifert, Orit Gressel, Raviv Peleg, Miek Jong

**Affiliations:** 1 Integrative Oncology Program, The Oncology Service, Zebulun Medical Centers, Clalit Health Services, Haifa and Western Galilee District, Carmel &, Lin, Israel; 2 Rappaport Faculty of Medicine, Technion-Israel Institute of Technology, Haifa, Israel; 3 Center for Integrative Complementary Medicine, Faculty of Medicine, Shaare Zedek Medical Center, Hebrew University of Jerusalem, Jerusalem, Israel; 4 Department of Pediatrics, Division of Oncology and Hematology, Charité - Universitätsmedizin Berlin, Corporate Member of Freie Universität Berlin, Humboldt-Universität Zu Berlin, and Berlin Institute of Health, Berlin, Germany; 5 Pediatric Hematology Oncology, Schneider Children’s Medical Center of Israel, Integrative Pediatric Medicine Program, Petach Tikva, Israel; 6 Department of Community Medicine, Faculty of Health Sciences, The Arctic University of Norway, National Research Center in Complementary and Alternative Medicine (NAFKAM), UiT, Tromsø, Norway

**Keywords:** Integrative oncology, Pediatric oncology, Pediatric hemato-oncology, Integrative medicine, Complementary alternative medicine

## Abstract

**Purpose of the review:**

Children and adolescents with cancer, along with their parents and other informal caregivers, often report using complementary and alternative medicine (CAM) during active oncology and hemato-oncology treatment. Some adopt an “alternative” approach to conventional medical care, which often entails the use of these practices without the knowledge of the treating pediatrician. In contrast, many others search for consultation provided by a pediatric integrative oncology (IO) practitioner working with the conventional medical team. IO seeks to provide evidence-based complementary medicine therapies, many of which have been shown to augment conventional supportive and palliative care, while ensuring the patient’s safety. The present narrative review examines the current state of and future direction for the IO setting of care.

**Recent findings:**

A large body of published clinical research supports the effectiveness of leading Pediatric IO modalities, while addressing potential safety-related concerns.

**Summary:**

Despite the growing amount of clinical research supporting the beneficial effects and implementation of Pediatric IO models of care, there is still a need for further studies in order to establish clinical guidelines in the treatment of children and adolescents with cancer. Such IO-directed guidelines will need to address both the effectiveness and the safety of the CAM modalities being used in pediatric oncology and hemato-oncology settings, promoting a better understanding among pediatric healthcare professionals and helping them understand the indications for referral to the IO treatment service.

## Introduction

The past two decades have seen significant advances in the treatment of pediatric patients with cancer, though a number of challenges and uncertainties remain. These include diagnostic and therapeutic obstacles, as well as negative healthcare-related experiences in the past which may increase the interest of a pediatric patient, their family, and their oncology healthcare provider (HCP) to the use of complementary and alternative medicine (CAM). The modalities are often being provided within an “alternative” context of care, or a “complementary” setting when used in parallel with conventional care. The National Cancer Institute has defined CAM as medical products and practices which are not currently part of standard medical care [1]. Integrative oncology (IO) differs from CAM in that it contextualizes the practice of evidence-based complementary medicine modalities within the conventional oncology setting, as an integral part of supportive and palliative care services being provided to patients. The Society for Integrative Oncology (SIO) defines integrative oncology as a “patient-centered, evidence-informed field of cancer care that utilizes mind and body practices, natural products, and/or lifestyle modifications from different traditions alongside conventional cancer treatments” [2]. The SIO goals for IO are “to optimize health, quality of life, and clinical outcomes across the cancer care continuum and to empower people to prevent cancer and become active participants before, during, and beyond cancer treatment”. Tortora et al. have published a comprehensive definition for pediatric IO, through an international consensus highlighting the need to incorporate IO modalities and lifestyle modifications within a collaboration with oncology HCPs [3•]. Over the past five years, a number of pediatric IO programs have been established in Germany [4, 5] and Brazil [6].

Another related therapeutic approach is that of *traditional medicine*, defined by the World Health Organization (WHO) as “knowledge, skill, and practices based on the theories, beliefs, and experiences indigenous to different cultures, whether explicable or not, used in the maintenance of health as well as in the prevention, diagnosis, improvement or treatment of physical and mental illness” [7]. Traditional medicine use is highly popular in low-and middle-income countries, as well as among patients with a high affinity to herbs, nutrition (“food as therapy” [8]) and other traditional medicine modalities [9]. A study from northern Israel found that while Jews and Arabs had similar rates of CAM use, patients from a higher socioeconomic status reported using primarily non-herbal supplements and homeopathy; and those from lower-to-middle socioeconomic status more likely to use traditional Arab medicine, mainly herbs [10]. In Guatemala City, where traditional medicine and CAM are reportedly being used by 90% of parents of children with cancer, two-thirds of respondents reported using these modalities in conjunction with conventional treatment for supportive care-related indications, suggesting a complementary medicine context of care [11]. Complementary medicine practices are prevalent in Turkey and Malysia, where they are being used together with conventional treatment [12, 13]. In Kenya, many HCPs are supportive of CAM use, despite the fact that only 18% of HCPs interviewed had a positive attitude toward traditional medicine and CAM; and 56% reported a belief that the combination with chemotherapy is “the best way to cure cancer” [14].

Rates of CAM use in the pediatric cancer population range from 6 to 91% [15•], and take place within a broad range of clinical settings and with modalities which vary according to the setting and culture. In Canada, 60% of pediatric oncology patients have been reported to be using CAM, primarily multivitamin use [16]; 64% in the US, primarily prayer, supplement use and massage [17]; 69% in Switzerland, predominantly homeopathy [18]; and 87% in France, mainly homeopathy, chiropractic, and faith-healing [19]. Rates of CAM use during survivorship range from 52% (Hong-Kong) to 58% (US), with younger adults more likely to choose manipulative, body- and mind–body therapies than younger children [20, 21]. The prevalence of CAM use in palliative care settings ranges from 29% in Canada [22]), 43% in Germany [23•] and 63% in Hong-Kong [24].

Expectations among patients and their parents from CAM include improving general wellbeing; 'strengthening' the immune system; and reducing adverse effects of cancer treatments [25, 26]. Providing a “cure” and improving survival or preventing recurrence are mentioned as well, though less frequently (between 5- to 49%, depending on the country and setting) [27–29]. Expectations among parents that CAM treatment can “cure” their child’s cancer, may contrast to those of the treating HCP [30]. In as many as 65% cases the use of CAM is not being disclosed to the patient’s pediatric oncology HCP [31, 32]. Non-disclosure of CAM use by parents of pediatric oncology patients has been attributed to their fear of a negative reaction from the oncologist [33•].

In contrast to the frequent non-disclosure of CAM use to HCPs, pediatric IO programs are seen as a positive addition by HCPs who emphasize open and non-judgmental communication with children and parents, identifying treatment goals and referring to CAM modalities for quality of life (QoL)-related concerns [34–36]. At the same time, these HCPs frequently do not provide a structured referral to a IO consultation or to CAM providers outside the hospital setting [37]. This has been attributed to the lack of knowledge among HCPs on the potentially beneficial effects of IO in patient care [38, 39]. The lack of knowledge on this subject may be the result of the limited exposure CAM receives in the medical education curriculum, if at all, and the lack of defined graduate competencies [40, 41].

Clinical practices guidelines are important in providing information about IO to HCPs on the available evidence supporting the use of these therapies in patient care. These include the clinical practice guidelines published by the Society for Integrative Oncology (SIO) and the American Society for Clinical Oncology (ASCO) for integrative oncology in the adult cancer population [42–44], though guidelines for pediatric patients with cancer are currently not available. Nevertheless, systematic reviews published in recent years summarize the effectiveness and safety of specific CAM modalities for cancer-related symptoms in the pediatric patient population, including for pain [45], nausea and vomiting [46] and other adverse effects of anti-cancer treatment [47, 48].

This is, to the best of our knowledge, the first narrative review on the published research on CAM across the pediatric cancer care trajectory. The review set out to highlight the potential for certain CAM modalities which should be provided in the pediatric IO setting, in conjunction with conventional anti-cancer treatment during survivorship and palliative/end-of-life care. The findings of the review will hopefully inform oncology HCPs, pediatric patients and their families, providing researchers with a foundation enabling the creation of clinical practice guidelines for the pediatric IO setting.

## Methods

A multi-disciplinary team of six researchers from Israel, Norway, and Germany undertook the design of the present narrative review: four physician-researchers, one non-MD researcher, and one non-MD practitioner who also represents a parent advocacy perspective. The design of the review was guided by key elements identified in the narrative review by Sukhera [49], while using items of quality assessment from a narrative review by Baethge et al. [50] The following review questions were formulated:

1. Which CAM modalities are potentially effective during cancer treatment, survivorship and in the palliative stage of pediatric cancer care?
2. Which CAM modalities are safe to use during cancer treatment; throughout survivorship; and in the palliative stage of pediatric cancer care?
3. Which evidence-based recommendations can be made for the integration of CAM modalities in pediatric oncology care?

A search was then conducted, using MEDLINE/Pubmed, the Cochrane Database of Systematic Reviews, and the Memorial Sloan-Kettering Integrative Medicine Service database. Keywords searched for this purpose included alternative/ complementary/traditional/integrative medicine; pediatric oncology/hemato-oncology; palliative care and survivorship; doctor-patient communication and disclosure; bone marrow transplantation; effectiveness, safety/risk; and other CAM modality-specific keywords (e.g., acupuncture, touch, mind–body, herbal medicine, nutrition, Anthroposophic medicine). Explanatory studies (i.e., randomized, controlled trials, RCTs); quasi-experimental studies; n = 1 RCT studies; meta-analyses and systematic reviews were examined.

Included studies examined CAM interventions in children and adolescents up to 18 years of age currently diagnosed with or surviving cancer. Only peer-reviewed studies published in the English language were included, and limited to CAM modalities categorized under one of four domains: herbal medicine (including homeopathic remedies and aromatherapy), non-herbal dietary supplements, manual therapies (including acupuncture), and mind–body therapies. Data on the study design, intervention, comparators, patient populations, outcomes and safety were extracted and narratively described in text, and structured in supplemental tables. Recommendations were made by the authors for Question 3 (evidence-based recommendations supporting CAM modalities in pediatric oncology care), based on the available literature and within the context of their personal, scientific, clinical and medical education-related experiences. The results of the review were presented in accordance with the Narrative Review Checklist, published by Green et al. [51]

### CAM in Active Cancer Treatment

Most included studies focused on potential beneficial effects of CAM for adverse effects of cancer treatment, including chemotherapy-induced nausea and vomiting, neutropenia, oral mucositis, and stomatitis. The research also addressed the ability of CAM to relieve post-operative distress and pain. The research supporting the beneficial and safe use of CAM treatment groups during active cancer treatment are summarized in Table 1.

**Table 1:** Characteristics of included studies on CAM in pediatric cancer treatment

Intervention	Study design	Study population	Outcomes*	Risk/Safety	Reference
Herbal medicine					
Honey *2.5 g/kg body weight per honey dose twice weekly for 12 weeks*	Double-blind RCT comparing honey or olive oil	A total of 42 children with leukemia suffering from oral mucositis	Less severe oral mucositis and pain in children receiving Manuka honey. Less pain in children receiving olive oil	No gastrointestinal adverse effects or an allergic reaction. Olive oil reported with bad taste	^52^
	Randomized crossover clinical trial with two arms (honey vs. control)	A total of 40 children (age 2.5–10 yrs.) with acute lymphoblastic leukemia	Decreased febrile neutropenia (FN) episodes, the number of patients admitted with FN and the duration of hospital stay. Also, honey consumption improved the levels of hemoglobin	Did not produce any serious side effect	^53^
Zingiber officinale (Ginger powder) *20–40 kg: 167 mg capsules X 6* *40–60 kg: 400 mg capsules X 5*	Double-blind placebo RCT	Children & young adults receiving high emetogenic chemotherapy for bone sarcoma	Reducing severity of acute and delayed CINV as additional therapy to ondansetron and dexamethasone	Ginger was well-tolerated. No significant adverse effects	^54^
Matricaria chamomilla (Chamomile) *2.5 ml of either chamomile or placebo syrup*	Triple-blind placebo RCT with two arms (chamomile vs. control)	A total of 40 patients (age 2–18 yrs.) with acute lymphoblastic leukemia	An increasing trend of absolute neutrophil count vs. decreasing trend in placebo group	No serious side effects were reported	^55^
Huangqi (Astragalus membranaceus) *Huangqi injection (0.5 mL/kg·d)*	Double-blind placebo RCT	A total of 91 children with acute lymphoblastic leukemia during remission induction chemotherapy	Lower incidence and shorter duration of infection; higher neutrophil count after chemotherapy; lower incidence rates of respiratory tract infection, urinary tract infection, blood infection, and skin and soft tissue infections	Not reported in the abstract	^59^
Herbs used in aromatherapy					
Zingiber officinale (Ginger inhalation) *4 drops of essential oil prior and during chemotherapy infusion*	Double-blind placebo RCT with three arms (ginger versus water versus shampoo	A total of 49 patients with varied types of cancer, undergoing emetogenic chemotherapy (age 8 to 21 yrs.), n = 20 (ginger), n = 10 (water), n = 19 (shampoo)	Ginger did not significantly decrease nausea compared to the water and shampoo placebo groups	Ginger was well-received, well-tolerated and not toxic	^56^
Non-herbal dietary supplements					
*Oral, 6 g/m2 per dose twice daily, maximum 10 g/dose* *Parenteral, 0.4 g/kg/day for 3 days* Glutamine *Oral, 400 mg/kg body weight per day* *Enteral, 0.65 g/kg/day for 7 days*	Double-blind placebo RCT	A total of 56 patients (age 5–21 yrs.) schedule for treatment with vincristine for leukemia, lymphoma, extracranial solid tumor/medulloblastoma	Higher number of children progressed on the sensory neuropathy scale in the placebo compared to the glutamine group. Children receiving glutamine rated their quality of life higher	Glutamine was well-tolerated; mild patient-reported side effects	^65^
	Retrospective, observational study	A total of 96 children with acute lymphoblastic leukemia (age 0–17 yrs), treated with high-dose methotrexate, with (n = 80) or without glutamine (n = 267)	The incidence of oral mucositis was significantly lower in the glutamine-treated group than in the control group (3.8% vs. 17.6%; p0.004)	No severe adverse effects were reported	^66^
	Double-blind placebo RCT	A total of 24 children with acute lymphoblastic leukemia (age 1–18 yrs.) who underwent consolidation phase chemotherapy and received high-dose methotrexate	Prevention of oral mucositis; Lower hospital stay duration and cost	Not reported	^67^
	RCT, cross-over trial	Patients (age 1–21 yrs.) undergoing treatment for malignancy and having at least two identical courses of chemotherapy along with (n = 19) or without glutamine (n = ?)	No significant changes in symptoms of mucositis were found Fewer children receiving glutamine required parenteral nutrition, with shorter duration of parenteral nutrition	No adverse effects attributed to glutamine were observed	^68^
*Topical, 100 mg twice daily* *Oral, 100 mg twice daily* *Topical, 200 mg daily* Vitamin E *Topical, 800 mg*	RCT with 2 arms (topical vs oral vitamin E)	A total of 63 children (age < 12 yrs) with chemotherapy-induced oral mucositis, receiving topical vitamin E (n = 30) or oral vitamin E (n = 33)	Topical application of vitamin E was significantly more effective than oral vitamin E in reducing chemotherapy-induced mucositis (p < 0.001)	Not reported	^69^
	RCT with 3 arms (vitamin E vs pycnogenol vs placebo (glycerine))	A total of 72 children (age 6–15 yrs) with chemotherapy-induced oral mucositis, receiving vitamin E (n = 24), pycnogenol (n = 24) or placebo (glycerine, n = 24)	Both vitamin E and pycnogenol were effective in the treatment of oral mucositis compared to placebo (P < 0.001)	Not reported	^70^
	Series of n = 1 double-blind RCTs	A total of 16 children (age ≥ 6 yrs) receiving repeated cycles of doxorubicin-containing chemotherapy, 45 postchemotherapy cycles were randomized to vitamin E (n = 22) or placebo (n = 23)	Topical vitamin E did not significantly prevent doxorubicin-induced oral mucositis compared to placebo	There was no observed toxicity associated with topical vitamin E administration	^71^
*Recommended daily intake per age group, for 30 days* Selenium *Recommended daily intake per age group, for 30 days*	Double-blind cross-over RCT (selenium versus placebo)	A total of 39 children (age ≤ 18 yrs) with leukemias, lymphoma and solid tumors receiving chemotherapy, randomized to selenium or placebo, and subsequent inversion	Supplementation with selenium reduced the side effects of chemotherapy such as fatigue, nausea, and impaired physical function. Renal and liver functions were also improved	Not reported	^76^
	Double-blind cross-over RCT (selenium versus placebo)	A total of 36 children (age ≤ 18 yrs) with leukemias, lymphoma and solid tumors receiving chemotherapy, randomized to selenium or placebo, and subsequent inversion	Supplementation with selenium reduced neutropenia in children with lymphoma and solid tumors	Not reported	^77^
*5* × *109 CFU twice daily, for 7 days* Probiotics & prebiotics *Oral or enteral, 1* × *108 CFU per day for about three weeks* *5* × *10*^*9*^* CFU twice daily for 7 days*	Systematic review	Inclusion: Three RCTs with a total of 177 children undergoing chemotherapy	Supplementation with prebiotics and probiotics may reduce chemotherapy-related side effects: nausea/vomiting, infection, and morbidity risk	Safe of these products was demonstrated in these three studies	^72^
	Single-blind RCT	A total of 60 children (age < 17 years) children with acute leukemia planned to receive chemotherapy, randomized to probiotics (n = 30) or placebo (n = 30)	Daily supplementation with probiotics reduced chemotherapy-induced gastrointestinal side effects in children with acute leukemia	Not reported	^74^
	Observational study of safety and feasibility	A total of 30 children (age 2–17 yrs) undergoing hematopoietic cell transplantation	Feasible to perform such as study	Administration was shown to be safe	^73^
	Double-blind RCT	A total of 113 children (age 5–15 yrs) with acute lymphoblastic leukemia, randomized to synbiotics (*n* = 58) or placebo (*n* = 55)	Reduced chemotherapy-induced diarrhea as well as rate of constipation and nausea and vomiting	Not reported	^75^
Homeopathy					
Homeopathic complex remedy *12 components with dilutions ranging from D2 to D6 potency*	Double-blind placebo RCT	A total of 32 patients (age 3–25 yrs.) undergoing allogeneic or autologous stem cell transplantation	May reduce significantly the severity and duration of chemotherapy-induced stomatitis	No significant difference between the groups in serious complications	^63^
Acupuncture					
Acupuncture *During either the second or third chemotherapy course*	Randomized multicenter crossover pilot trial	A total of 23 children receiving highly emetogenic chemotherapy randomized to acupuncture or no acupuncture	Lower need for rescue antiemetic medication and episodes of vomiting per course	Pain from needling	*
Laser acupuncture *using a visible Class II red laser during the first day of chemotherapy cycle*	Single-blind RCT comparing active vs. inactive laser acupuncture	A total of 17 children & adolescents receiving medium-to-highly emetogenic chemotherapy agents	Relief of nausea within 5 days of chemotherapy; reduced number of episodes of vomiting on days 2 and 3 after chemotherapy	Not reported	**
Acupressure and manual therapies					
Manual and wristband acupressure	Double-blind placebo RCT	A total of 44 patients (age 5–18 yrs.) randomized to (a) manual acupressure before the first chemotherapy, followed by placebo manual acupressure before the next chemotherapy; (b) wristband acupressure before the first chemotherapy, followed by placebo wristband acupressure before the next chemotherapy	Both manual and wristband acupressure reduce the severity and number of nausea and vomiting events; manual acupressure was more effective in reducing the severity and number of nausea and vomiting events when compared to wristband acupressure	Not reported in the abstract	^82^
Massage *3 massage session/week over a one-week period*	Single-blind placebo RCT	A total of 52 patients (age 10–18 yrs.) hospitalized in a pediatric cancer ward were randomized to massage vs. usual care	Reduced interference of pain in walking and decrease the intensity of the pain experienced by the child	Not reported	^83^
Healing touch *30 min at each inpatient or outpatient encounter*	RCT	A total of 15 patients (age 3–18 yrs.) in pediatric oncology inpatient and outpatient units were randomized to healing touch intervention vs. reading/play activity (controls)	Decrease scores for pain, stress, and fatigue. Parents' perception of their children's pain decreased significantly	Not reported	^85^
Mind–Body therapies					
Hypnosis *15-to-30-min session involving imagination fantasy during chemotherapy administration*	Single-blind placebo RCT	A total of 20 pediatric patients undergoing chemotherapy randomized to hypnosis or standard treatment	Decreased antiemetic medication usage and reduced anticipatory nausea during chemotherapy	Not reported in the abstract	^88^
	RCT	A total of 54 pediatric patients undergoing chemotherapy, randomized to hypnosis, non-hypnotic distraction/relaxation, or attention placebo (control)	Shorter duration of nausea and vomiting	Not reported	^89^
Mindfulness-based stress reduction (MBSR) with music therapy *Eight sessions of 13 h total duration*	RCT	A total of 101 patients (age 10–21 yrs.) diagnosed with osteosarcoma were randomized to MBSR combined with music therapy or control group with no psychological intervention	Reduce pain and anxiety and improved the quality of sleep	Not reported	^90^
Multimodal symptom-management program *including progressive muscle relaxation and, guided imagery recommended to be practiced at least once a week during each cycle of chemotherapy*	Exploratory pilot randomized study with qualitative interview	A total of 50 patients (age 10–18 yrs.) undergoing chemotherapy randomized to symptom-management program plus usual care or usual care (controls)	Significantly less fatigue. No differences regarding nausea and vomiting, pain, mucositis or anxiety between groups	Both children and parents reported a positive experience	^91^

QoL, quality of life, CINV, chemotherapy-induced nausea and vomiting; IO, integrative oncology, MBSR, mindfulness-based stress reduction

^*^Data based only on significant (p < 0.05) results

^*^Gottschling S, Reindl TK, Meyer S, Berrang J, Henze G, Graeber S, Ong MF, Graf N. Acupuncture to alleviate chemotherapy-induced nausea and vomiting in pediatric oncology—a randomized multicenter crossover pilot trial. Klin Padiatr. 2008 Nov-Dec;220(6):365–70. https://doi.org/10.1055/s-0028-1086039. Epub 2008 Oct 23. PMID: 18,949,672

^**^Varejão CDS, Santo FHDE. Laser Acupuncture for Relieving Nausea and Vomiting in Pediatric Patients Undergoing Chemotherapy: A Single-Blind Randomized Clinical Trial. J Pediatr Oncol Nurs. 2019 Jan/Feb;36(1):44–54. https://doi.org/10.1177/1043454218810140. PMID: 30,798,684

### Herbal Medicine

The use of herbs and other dietary supplements is popular among children with cancer, though the research supporting this practice is limited, at best (Table 1). Manuka honey demonstrated a beneficial effect in the relief of oral mucositis; and olive oil for pain in children with leukemia [52]. Bee honey (considered an herbal remedy in traditional Greco-Arab medicine) was shown to decrease febrile neutropenia in children with acute lymphoblastic leukemia [53]. Other RCTs have shown that ginger (*Zingiber officinale*) can reduce acute and delayed chemotherapy-induced nausea and vomiting in children and young adults receiving highly emetogenic chemotherapy for bone sarcoma [54]; and Chamomile (Matricaria chamomilla) for neutropenia in children and adolescents with acute lymphoblastic leukemia [55]. Safety-related concerns were addressed in all the above studies, including adverse effects which were either mild or absent. In an RCT comparing essential ginger oil (aromatherapy) to placebo (water and shampoo) for chemotherapy-induced nausea, the ginger treatment was found to be safe and well-tolerated, though without beneficial effect on nausea scores in either treatment arm [56].

The research published on herbal medicine in the broader context of traditional medicine is extremely limited. Traditional systems using herbs are an integral part of TCM in China, Kampo medicine in Japan, Ayurvedic medicine in India, and Greco-Islamic medicine in the Middle East. In each system herbal products are chosen based on a structured diagnostic process, and are most often administered as multi-herbal formulas. An uncontrolled study in Egypt showed that black seed oil (Nigella Sativa), a leading Islamic medicinal herb mentioned in the holy Quran, decreased methotrexate-induced hepatotoxicity and improved survival in children with acute lymphoblastic leukemia [57].

In a systematic review examining patterns of TCM use in pediatric oncology patients, it was shown that most RCTs focused on hematological malignancies, using the herb Huangqi (Astragalus membranaceus), commonly used for treating myelosuppression, infection, and gastrointestinal concerns [58]. Huangqi injections were found to significantly reduce the incidence and duration of infection in chemotherapy-treated children with acute lymphoblastic leukemia, with higher neutrophil counts following treatment [59]. RCTs from China found that the combination of the Fuzheng Jianpi decoction with chemotherapy led to improved quality of life (QoL) and increased survival in children diagnosed with solid tumors [60]; and the addition of the Hewei Zhiou recipe to ondansetron hydrochloride helped relieve chemotherapy-induced vomiting among children with solid tumors [61].

Anthroposophic medicine is another system of integrative medicine which often includes herbal- and metal-derived remedies. A German multi-centered RCT found neither beneficial nor harmful effects with Anthroposophic supportive treatment for adverse effects of cancer therapies; or event-free survival in children aged 1–18 undergoing intensive-phase chemotherapy treatment [62].

Homeopathy differs from other CAM modalities in that it utilizes extremely high dilutions of herbal and other “mother” products. A study conducted in Israel of a complex homeopathic remedy (vs. placebo) showed reduced severity and duration of chemotherapy-induced stomatitis in patients aged 3–25 years undergoing allogeneic or autologous stem cell transplantation [63]. However, in a follow-up multi-centered trial, no statistically beneficial effect was found with the same homeopathic remedy, though a trend was found towards lower rates of narcotic analgesic use [64].

### Non-herbal Dietary Supplements

Several RCTs have investigated the use of non-herbal dietary supplements such as glutamine and vitamin E (tocopherol), as well as prebiotics and probiotics in the pediatric oncology setting (see Table 1). A study from the U.S. found that glutamine was well tolerated and associated with improved sensory function and overall QoL in children and adolescents undergoing vincristine treatment for leukemia, lymphoma, extracranial solid tumors and medulloblastoma [65]. Parenteral glutamine was shown to be safe and effective in reducing oral stomatitis in a retrospective observational study of children with acute lymphoblastic leukemia, when compared to controls receiving standard care alone [66]. An RCT of oral glutamine supplementation showed a reduced onset of oral mucositis, with a lower duration of hospitalization and expenditures in children and adolescents undergoing consolidation chemotherapy with high-dose methotrexate for acute lymphoblastic leukemia [67]. However, in another RCT among children with varied pediatric malignancies, no beneficial effect was found with oral glutamine in preventing chemotherapy-induced mucositis, though a reduced need for and duration of parenteral nutrition was observed in the oral glutamine-treated group [68].

Vitamin E has been shown to reduce the incidence of chemotherapy-induced oral mucositis in children with cancer (see Table 1). In one RCT, topically applied vitamin E significantly reduced the severity of chemotherapy-induced oral mucositis, when compared to oral administration of the vitamin[69]. In an RCT comparing oral vitamin E to the herbal formula pycnogenol, which contains pine bark, and a placebo preparation, both supplements significantly relieved grade 1-to-3 chemotherapy-induced oral mucositis [70]. However, in a series of n = 1, double-blind RCTs in doxorubicin-treated children, topical vitamin E was ineffective in the prevention of oral mucositis when compared to placebo [71]. Neither preparation of vitamin E (orally or topically administered) was associated with adverse effects.

A number of clinical studies (Table 1) have supported the effectiveness and safety of pre- and pro-biotic products. In a systematic review, these products were found to be safe and with a potential to reduce chemotherapy-induced nausea/vomiting and infections, with reduced morbidity [72]. The probiotic Lactobacillus plantarum was found to be safe and feasible for use in children and adolescents undergoing allogenic hematopoietic cell transplantation [73]. In a single-blinded RCT in children with acute leukemia, probiotic treatment was associated with a lower prevalence of chemotherapy-induced gastrointestinal symptoms, when compared to the placebo group. Three commonly reported gastrointestinal side effects—nausea, vomiting and abdominal distension—were shown to be significantly less prevalent in the probiotic-treated group [74]. Finally, in double-blinded RCT from Iran, a synbiotic formula (combination of prebiotics with probiotics) administered to children on maintenance chemotherapy for acute lymphoblastic leukemia, was associated with significantly lower rates of chemotherapy-induced diarrhea, constipation, nausea and vomiting [75]. All studies of pre- and probiotic supplement use showed this treatment to be both safe and well-tolerated by patients.

Other non-herbal supplements examined in the pediatric oncology setting include two double-blind RCTs showing a beneficial effect with selenium, an essential dietary trace mineral, in children with leukemia, lymphoma, and solid tumors undergoing chemotherapy (Table 1). In the first RCT, a beneficial effect was found for chemotherapy-induced fatigue and nausea, as well as renal and liver function [76]. In the second study, patients treated with selenium had lower rates of neutropenia [77].

### Acupuncture and Manual Therapies

Acupuncture treatment entails the insertion of fine, sterile and single-use needles into acupuncture points which have been selected based on the principles of TCM, or else the findings of clinical research (medical acupuncture). The research examining the effectiveness of acupuncture in the pediatric IO setting is extremely limited, and was not addressed in the SIO/ASCO clinical guidelines on integrative medicine for cancer-related pain, anxiety and depression in the adult population [78, 79]. A previous meta-analysis of five RCTs (241 oncology patients) treated with acupuncture/acupressure (intervention, 119; controls, 122), concluded that acupuncture and related techniques can significantly reduce chemotherapy-induced nausea and/or vomiting, when compared to control interventions (Table 1). Three RCTs which addressed the safety of acupuncture reported minor adverse effects, such as pain from needling or acupressure bands that were too tight [80]. A systematic review concluded that despite a high risk for potential bias and low number of studies, there is nevertheless preliminary evidence supporting the effectiveness of acupuncture and limited evidence for acupressure in alleviating nausea and vomiting in pediatric oncology patients [81].

Acupressure entails the application of localized pressure on acupuncture points, with manual and wristband acupressure (vs. a “placebo touch” intervention) shown to reduce the severity and frequency of chemotherapy-related nausea and vomiting in a Turkish study of children and adolescents receiving chemotherapy [82].. An RCT from Portugal found that massage therapy reduced the interference of walking-associated pain in a pediatric cancer population, while contributing to the relief of pain and its impact on their daily activities [83]. In a small feasibility study conducted in pediatric oncology inpatient and outpatient units in Hawaii, patients were randomized to “Healing Touch” vs. controls experiencing reading/play activity. Patients in the intervention arm reported better scores for pain, stress and fatigue, as reflected in the parents' perception of their child's pain [84]. Foot reflexology was also shown to have a significant effect on reducing pain and improving physiological parameters in children with leukemia undergoing intrathecal chemotherapy treatments [85].

### Mind–Body Therapies

Mind–body medicine includes a wide range of therapeutic options, including relaxation, deep breathing, meditation, hypnosis, mindfulness and spiritual modalities, some of which are based on Eastern philosophy (e.g., Buddhism, Hinduism, Daoism) or religious practices (e.g., Islamic medicine, tribal medicine), including healing, prayer and blessings which are highly prevalent among Greek parents of children with cancer [86]. Some mind–body modalities overlap with movement therapies (e.g., breathing and mindfulness aspects in Yoga or Chi Gong) and manual/touch modalities (e.g., gestures of presence during Anthroposophic nursing manual therapies, acupressure, Shiatsu, or acupuncture). Often, classical mind–body interventions, such as guided imagery or hypnosis, are provided together with a manual therapy (e.g., reflexology, acupuncture, or acupressure), especially in the integrative oncology setting [87].

A U.S. study found evidence supporting the use of hypnosis for chemotherapy-induced nausea and vomiting, reducing “as-needed” use of antiemetic medications and less frequent anticipatory nausea when compared to controls [88]. In an earlier RCT, a greater reduction of both anticipatory and post-chemotherapy symptoms was observed in children treated with hypnosis, compared to a non-hypnotic distraction/relaxation intervention (with only a maintenance effect) and a third group of controls (attention placebo), who showed worsening of their symptoms over time [89].

An RCT from China found reduced pain and anxiety, as well as improved sleep quality with a combined regimen of mindfulness-based stress reduction (MBSR) and music therapy in patients with osteosarcoma (aged 10 to 21 years) [90]. A second RCT from Singapore examined a multi-modal approach in children and adolescents, from cancer diagnosis to treatment, comparing home-based interventions (including progressive muscle relaxation and guided imagery) to usual care. While fatigue was significantly improved with the intervention, no between-group differences were found regarding nausea and vomiting, pain, mucositis or anxiety. However, both children and parents in this group reported a positive experience from the study interventions [91].

A pilot RCT from Canada demonstrated the feasibility and acceptability of a sleep intervention (sleep hygiene combined with deep breathing and progressive muscle relaxation) for children undergoing maintenance chemotherapy for acute lymphoblastic leukemia, finding no statistically significant benefits [92]. In another study of children undergoing stem cell transplantation, a mixed model approach with randomization to a child-targeted intervention involving massage and humor therapy; additional parent intervention involving massage and relaxation/imagery; or standard care was explored [93]. No beneficial effects were found regarding somatic distress and mood disturbance, or for length of hospitalization and use of narcotic analgesic and antiemetic medications. In another RCT conducted in a hematopoietic stem cell transplantation setting, individualized yoga was explored for feasibility in children receiving intensive chemotherapy, here too with no effectiveness found with respect to the study’s outcomes [94]. In a pediatric stem cell transplantation setting in the U.S., patient-parent dyads were randomized to a child intervention with massage and humor therapy; the same intervention, plus massage and relaxation/imagery provided by the parent; or standard care [95]. No significant differences across treatment arms were observed regarding parental distress, global adjustment outcomes of depression, frequency of PTSD, or any other beneficial outcomes with the interventions.

### CAM in Survivorship

Pediatric cancer survivors frequently suffer from long-term and late effects related to their cancer and/or its treatment [96–98]. These often include fatigue and memory/learning difficulties, as well as general psychological distress. Survivors are at risk for developing cardiovascular disease, hormonal and immune deficiencies, and in some cases even secondary malignancies [99, 100]. Impaired QoL can affect daily functioning long into adulthood [101], highlighting the importance of providing these survivors with supportive care during the post-treatment period. Most of the research on this patient population has been focused on lifestyle changes, including technology-assisted interventions such as e-health to support physical activity and a healthy diet [102, 103]. Despite the widespread use of CAM among childhood cancer survivors [104], the research on the potential effectiveness and safety of these modalities for ongoing QoL-related concerns is limited [105]. A study from China examined an educational program promoting the safe and effective use of TCM among childhood cancer survivors, addressing the implications of referring them to practitioners working with the cancer survivor community [106].

Mindfulness-based interventions have been shown to be a promising intervention during this period, addressing emotional stress and improving QoL among adolescent cancer survivors. In a Belgian study, adolescent and young adult cancer survivors reported a significant reduction in emotional distress following an 8-week MBSR intervention, with improved QoL at 3 months and a significant reduction in negative attitudes toward self on cognitive vulnerability parameters [107]. In another study from the U.S., adolescent and young adult sarcoma survivors responded positively to a mobile-based mindfulness and social support program, though no significant psychological functioning parameters were found to improve [108]. In another U.S. study, the feasibility of a yoga program for children and adolescents during survivorship found a significant decrease in anxiety scores [109].

A scoping review mapping the possible benefits and risks of wilderness therapies found that spending time in nature may increase social involvement, self-esteem, self-confidence, self-efficacy, social support and physical activity of childhood, adolescent and young adult cancer survivors [110]. A number of wilderness programs (including mind–body exercise) have been developed in the U.S. and Sweden to specifically meet the needs of adolescents after cancer treatment. A preliminary evaluation of these programs found them to be safe and of interest to pediatric cancer survivors, potentially improving physical functioning, anxiety, depression, fatigue, peer relations, nature-connectedness and QoL [111–113].

### CAM in Palliative Care

A number of systematic reviews have explored the effectiveness of palliative pediatric care in pediatric palliative care and home-based services [114, 115]. One review assessed the effectiveness of CAM therapies in the management of symptom clusters in children and adolescents undergoing palliative cancer care [116]. Of the five quasi-experiments meeting the review’s eligibility criteria, three studies found a significantly beneficial effect with therapeutic massage and Reiki in addressing the pain-anxiety-worry-dyspnea cluster. A pilot study from Canada found an immediate decrease in pain and worry with massage provided during palliative cancer care, though the long-term effects of this intervention were unclear [117].

### Parent and Informal Caregiver Perspectives

The health-belief model of care among parents and guardians of children with cancer often includes decision-making based on personal experience with CAM, religious beliefs and recommendations from family members and friends [118]. In contrast, health-belief models of care among HCPs are invariably oriented first and foremost to evidence-based research and published clinical guidelines [119]. A qualitative study from the U.S. found that many pediatric oncologists report a need to “negotiate” with parents about CAM use, addressing issues such as delaying or refusing anti-cancer treatment; non-adherence to conventional treatment regimens; or even stopping treatment altogether [120]. A national survey of parents of children with cancer in Germany found that a lack of trust in conventional medicine was rarely associated with increased use of CAM [121]. This may reflect more of a practical approach than an “alternative” paradigm of care, as can be seen by an increased use of CAM following failure of first-line anti-cancer therapy [122], or during palliative or end-of-life-care [123, 124]. At the same time, parents are less likely to consider CAM as the time from relapse of the disease progresses [125].

The research examining the impact of the pediatric IO model on the treatment of parents or other informal caregivers of children with cancer is extremely limited. A University of Minnesota School of Nursing study explored the impact of massage in children-parent dyads, concluding that the intervention was more effective than “quiet time” at reducing heart rate in children; anxiety in younger children (< 14 years); and anxiety among parents [126]. Spiritual interventions, reflecting the more religious pole of mind–body modalities, were examined in two RCTs conducted in Iran. In the first study, Borjalilu et al. randomized 42 mothers of children diagnosed with cancer to a spiritual care training or a control group [127], with a significantly greater effect found on anxiety and spiritual-religious scores in the intervention group. In the second study, Ahmadi et al. found a significant reduction of anxiety among mothers of children with cancer who underwent a 20-min, 3-day writing technique intervention addressing their desires, wishes and expectations from Allah, when compared to controls whose writing described only their normal daily schedule [128]. These limited findings should encourage further research examining the potential role of IO in addressing concerns among parents and other informal caregivers of children with cancer. The goals of this research should address quality of life-related concerns among the child’s parents (e.g., anxiety, pain, fatigue and appetite loss), as well as improving coping, functioning, and resilience along the journey of pediatric cancer.

## Clinical Implementation

While the evidence supporting the role of pediatric IO is still limited, the findings presented in this review indicate that they should be considered, as part of the supportive and palliative care the patient is receiving. The following aspects regarding the establishment and running of a IO program within the pediatric oncology/hemato-oncology setting should be included:

1. **Setting goals**: Focus primarily on QoL-related concerns for frequently reported adverse effects of conventional anti-cancer treatments, especially those for which current supportive/palliative care options are of limited effectiveness (e.g., cancer-related fatigue, nausea, stomatitis). It is important to clarify that these are the program’s goals, as opposed to what may be expected by the child’s parents who may have an “alternative” approach to CAM, as a replacement for conventional treatment while providing a “cure” for their child’s cancer.
2. **Promoting effective communication:** Provide training to the Pediatric IO team which includes communication competencies with respect to children, parents and siblings. This is especially true, though not exclusively, for those expressing an “alternative” health-belief model, with a high affinity to alternative and traditional medicine within the context of religious, spiritual, and cultural beliefs and values. Training should address language and cultural barriers, while promoting communication with other healthcare providers to enhance the multi-disciplinary team collaboration.
3. **Safety First**: Safety is a key issue which needs to be addressed in the Pediatric IO setting. This relates not only to the potential for adverse effects of CAM interventions, as well as negative interactions with oncology treatments, but also to the need for awareness among the patient’s oncology HCPs that they are using these therapies in conjunction with conventional anti-cancer treatments [129••]. Pediatric IO services should include an integrative oncology HCP in the pediatric oncology/hemato-oncology team. This may reduce the risks associated with the use of unmonitored and unproven CAM therapies outside the conventional oncology setting. Integrative oncology HCPs are trained in and thus able to participate in the ‘two worlds’ of evidence-based conventional and CAM paradigms of care, communicating with patients, parents and informal caregivers, including those with a high affinity to alternative and traditional medicine. They are often able to prevent delays in diagnosing the cancer; address issues related to avoidance and adherence to integrative care; and, in some cases, facilitate the utilization of the available palliative care services [130, 131]. It is also extremely important to encourage safety/risk documentation during IO assessments, including for modalities considered safe (see Table 1 for examples of these risks). At the same time, it is important that CAM-associated risks in the Pediatric IO setting are not over-diagnosed, but held to the same standard as conventional medical interventions.
  - In addition to the above points, it is important to provide training to pediatric IO consultants, who can then proactively provide guidance and advice on the safe use of herbal and dietary supplements, while ensuring that potential interactions with conventional treatments are prevented. Pediatric IO practitioners must also be knowledgeable in the clinical settings of pediatric oncology, including working in sterile conditions with bone marrow transplant patients; or treating a child at risk for a line infection (e.g., central venous port/picc lines; nasogastric feeding tubes; indwelling urinary catheters; etc.).
4. **Specific challenges in pediatric oncology care**: Address considerations specific to the pediatric-oncology setting, such as extended hospitalization requiring bed rest, which can increase the risk of acute vascular thrombosis; and emotional and psychological distress. In the bone marrow transplant setting, the risk for graft-versus host disease (GVHD) is high, leading to both physical and emotional stress on the pediatric patient, parents and family members, as well as on the oncology HCP.
5. **Effectiveness of the CAM intervention**: Review the scientific literature to ensure that the CAM interventions being used are evidence-based regarding effectiveness; and that they provide a significant added value to conventional treatment, addressing unmet concerns of the child/adolescent and their parents. Table 2 presents pediatric IO modalities which can be considered for specific pediatric oncology treatment-related toxicities.
6. **Research and academic activities**: Encourage clinical research in the clinical pediatric IO setting, including its role in supportive and palliative care, focusing on mental well-being and quality of life. Use explanatory (i.e., RCTs) and pragmatic (i.e., uncontrolled, observational studies) methodologies to assess the impact of the program, and to identify real and potential barriers to its implementation. At the same time, create medical education initiatives, acquainting medical students and nurses with the patient-centered and treatment-tailored CAM approach, within a multidisciplinary context of care involving pediatrics, oncology/ hematology, nursing, palliative care, and psycho-oncology.

**Table 2:** Integrative medicine modalities to consider using for specific pediatric oncology treatment-related toxicities

Quality of life- related concerns (or oncology treatment-related toxicity)	Intervention	Study population	Study design	Potential beneficial outcomes	Risk considerations	Reference
Gastro-intestinal related concerns						
Oral mucositis	Honey	Children with leukemia suffering from oral mucositis	Double-blind RCT comparing honey or olive oil	Less severe oral mucositis and pain	Olive oil reported with bad taste	^52^
	Glutamine	Patients with acute lymphoblastic leukemia treated with high-dose methotrexate	Double-blind placebo-controlled RCT	May prevent oral mucositis; Lower hospital stay duration and cost	Not reported	^67^
	Vitamin E	Chemotherapy-induced oral mucositis in children and young adolescents	RCT comparing topical vs oral vitamin E; RCT comparing vitamin E vs pycnogenol vs placebo	Topical vitamin E is more effective than oral in reducing chemotherapy-induced mucositis; vitamin E and pycnogenol may be effective compared to placebo	Not reported	^69,70^
Chemotherapy induced nausea and vomiting (CINV)	Zingiber officinale (Ginger)	Patients with bone sarcoma receiving highly emetogenic chemotherapy	Double-blind RCT	Reducing severity of acute and delayed CINV as add-on therapy to ondansetron and dexamethasone	Well-tolerated	^54^
	Acupuncture	Children receiving highly-emetogenic chemotherapy	RCT	Lower need for rescue antiemetic medication and fewer episodes of vomiting per course	Pain from needling	*
	Laser acupuncture	Patients undergoing moderately-emetogenic chemotherapy	Single-blind RCT comparing active vs. inactive laser acupuncture	May relieve nausea within 5 days of chemotherapy and reduce vomiting episodes on days 2 and 3	Not reported	**
	Acupressure	Patients treated before the first and second chemotherapy cycles	Double-blind placebo RCT comparing manual vs. placebo vs. wristband acupressure	Manual acupressure is more effective than wristband acupressure in reducing the severity and number of nausea and vomiting episodes	Not reported	^82^
	Hypnosis	Patients undergoing chemotherapy	RCTs comparing hypnosis, non-hypnotic distraction/relaxation, or attention placebo; or hypnosis vs. standard treatment	May reduce anticipatory nausea, shorten duration of nausea and vomiting, and decrease antiemetic medication usage during chemotherapy	Not reported	^88,89^
Diarrhea and constipation	Probiotics & prebiotics	Children undergoing chemotherapy including for acute lymphoblastic leukemia	Systematic review; Single-and double- blind RCTs	May reduce chemotherapy-induced diarrhea, constipation and CINV	Considered generally safe	^72,74,75^
Pain and neuropathy						
Chemotherapy-induced peripheral neuropathy (CIPN)	Glutamine	Vincristine-induced peripheral neuropathy in patients treated for leukemia, lymphoma and medulloblastoma	Double-blind placebo RCT	May be beneficial for reducing sensory neuropathy progression	Well-tolerated; mild patient-reported side effects	^65^
Pain	Massage	Hospitalized patients in a pediatric cancer ward	Single-blind placebo RCT comparing 3 massage sessions/week vs. usual care	May reduce the interference of pain in walking and the child’s pain-perceived intensity	Not reported	^83^
	Healing touch	Patients admitted to inpatient and outpatient pediatric oncology units	RCT comparing healing touch vs. reading/play activity	May decrease pain, stress, and fatigue. Parents' perception of their children's pain decreased significantly	Not reported	^85^
Anxiety and sleep						
Anxiety	Mindfulness-based stress reduction (MBSR) with music therapy	Patients (age 10–21 yrs.) diagnosed with osteosarcoma	RCT comparing MBSR combined with music therapy or controls	May reduce anxiety, pain and improved sleep quality	Not reported	^90^
Fatigue						
Fatigue	Multimodal symptom-management program (incl. progressive muscle relaxation, guided imagery)	Patients (age 10–18 yrs.) undergoing chemotherapy	Randomized study with qualitative interview comparing symptom-management program plus usual care vs. usual care	May improve fatigue with no effect on nausea and vomiting, pain, mucositis, and anxiety	Positive experience reported by children and parents	^91^
Chemotherapy-related hematological toxicities						
Neutropenia	Honey	Children with acute lymphoblastic leukemia	Randomized crossover clinical trial	Decreased febrile neutropenia episodes and the duration of hospital stay. May increase hemoglobin level	No serious side effects were reported	^53^
	Matricaria chamomilla (Chamomile)	Patients with acute lymphoblastic leukemia	Triple-blind RCT	An increasing trend of absolute neutrophil count	No serious side effects were reported	^55^
	Astragalus membranaceus (Huangqi injection)	Children with acute lymphoblastic leukemia during remission- induction chemotherapy	Double-blind RCT	Higher neutrophil count following chemotherapy	Not reported in the abstract	^59^
	Selenium	Patients with leukemias, lymphoma and solid tumors undergoing chemotherapy	Double-blind cross-over placebo RCT	Selenium may reduce neutropenia	Not reported	^77^

QoL, quality of life

^*^Gottschling S, Reindl TK, Meyer S, Berrang J, Henze G, Graeber S, Ong MF, Graf N. Acupuncture to alleviate chemotherapy-induced nausea and vomiting in pediatric oncology—a randomized multicenter crossover pilot trial. Klin Padiatr. 2008 Nov-Dec;220(6):365–70. https://doi.org/10.1055/s-0028-1086039. Epub 2008 Oct 23. PMID: 18,949,672

^**^Varejão CDS, Santo FHDE. Laser Acupuncture for Relieving Nausea and Vomiting in Pediatric Patients Undergoing Chemotherapy: A Single-Blind Randomized Clinical Trial. J Pediatr Oncol Nurs. 2019 Jan/Feb;36(1):44–54. https://doi.org/10.1177/1043454218810140. PMID: 30,798,684

## Summary

The present narrative review presents the findings of much of the research on leading CAM modalities being used in the pediatric IO setting, with the goal of relieving symptoms and improving QoL among children and adolescents diagnosed with cancer. The review has a number of limitations include, first and foremost that it is not a systematic review with clearly defined criteria for inclusion of eligible studies. The inclusion of only English language papers may have excluded research in other languages which is relevant and important. Other limitations include the absence of a meta-analysis of the evidence provided, or a process of quality assessment of the studies that were chosen.

Nevertheless, the review does support the potentially beneficial effects for a number of CAM therapies in addressing QOL-related concerns among children and adolescents during active oncology/hemato-oncology treatment. The research on CAM for long-term and late effects during survivorship and palliative/end-of-life care is extremely limited. In addition, only a small number of the included studies addressed the safety of the CAM therapies examined, a major concern which needs to be addressed. Finally, the limited amount of research available on the pediatric IO model of care, in which CAM therapies are tailored to the pediatric patient with cancer while addressing the health-belief model and expectations of their parents and community, needs to be addressed as well. Further research of this model of care is of utmost importance, and should focus on the alleviation of QoL-related concerns among both children/adolescents; their parents and other relatives; and finally, the conventional HCPs responsible with their care.

## Data Availability

Not applicable.
